# Correlation of Body Mass Index and Proinflammatory Cytokine Levels with Hematopoietic Stem Cell Mobilization

**DOI:** 10.3390/jcm11144169

**Published:** 2022-07-18

**Authors:** Tso-Fu Wang, Yu-Shan Liou, Hsin-Hou Chang, Shang-Hsien Yang, Chi-Cheng Li, Jen-Hung Wang, Der-Shan Sun

**Affiliations:** 1 Department of Hematology and Oncology, Hualien Tzu Chi Hospital, Buddhist Tzu Chi Medical Foundation, Hualien 97002, Taiwan; tfwang@tzuchi.com.tw (T.-F.W.); kevinlcc1234@gmail.com (C.-C.L.); 2 Department of Medicine, College of Medicine, Tzu Chi University, Hualien 97004, Taiwan; hermann_yang@tzuchi.com.tw; 3 Buddhist Tzu Chi Stem Cells Center, Hualien Tzu Chi Hospital, Buddhist Tzu Chi Medical Foundation, Hualien 97002, Taiwan; 4 Department of Molecular Biology and Human Genetics, College of Medicine, Tzu Chi University, Hualien 97004, Taiwan; az0922663053@gmail.com (Y.-S.L.); hhchang@mail.tcu.edu.tw (H.-H.C.); 5 Department of Pediatrics, Hualien Tzu Chi Hospital, Buddhist Tzu Chi Medical Foundation, Hualien 97002, Taiwan; 6 Center of Stem Cell & Precision Medicine, Hualien Tzu Chi Hospital, Buddhist Tzu Chi Medical Foundation, Hualien 97002, Taiwan; 7 Department of Medical Research, Hualien Tzu Chi Hospital, Buddhist Tzu Chi Medical Foundation, Hualien 97002, Taiwan; jenhungwang2011@gmail.com

**Keywords:** body mass index, proinflammatory cytokines, hematopoietic stem cell mobilization, granulocyte colony-stimulating factor

## Abstract

This study investigated the correlation of body mass index (BMI) and proinflammatory cytokine levels with hematopoietic stem cell (HSC) mobilization triggered by granulocyte colony-stimulating factor (G-CSF). Stem cell donors (*n* = 309) were recruited between August 2015 and January 2018 and grouped into four groups according to their BMI: underweight (BMI < 18.5 kg/m^2^, *n* = 10), normal (18.5 kg/m^2^ ≦ BMI < 25 kg/m^2^, *n* = 156), overweight (25 kg/m^2^ ≦ BMI < 30 kg/m^2^, *n* = 102), and obese (BMI ≧ 30 kg/m^2^, *n* = 41). The participants were then administered with five doses of G-CSF and categorized as good mobilizers (CD34 ≧ 180/μL, *n* = 15, 4.85%) and poor mobilizers (CD34 ≦ 25/μL, *n* = 14, 4.53%) according to the number of CD34^+^ cells in their peripheral blood after G-CSF administration. The correlation between BMI and HSC mobilization was then analyzed, and the levels of proinflammatory cytokines in the plasma from good and poor mobilizers were examined by ProcartaPlex Immunoassay. Results showed that BMI was highly correlated with G-CSF-triggered HSC mobilization (R^2^ = 0.056, *p* < 0.0001). Compared with poor mobilizers, good mobilizers exhibited higher BMI (*p* < 0.001) and proinflammatory cytokine [interferon gamma (IFN-γ) (*p* < 0.05), interleukin-22 (IL-22) (*p* < 0.05), and tumor necrosis factor alpha (TNF-α) levels (*p* < 0.05)]. This study indicated that BMI and proinflammatory cytokine levels are positively correlated with G-CSF-triggered HSC mobilization.

## 1. Introduction

Hematopoietic stem cell transplantation (HSCT) has been performed for more than 50 years, since 1957 [1], to treat several hematological diseases and malignancies [2]. Bone marrow, umbilical cord blood, and granulocyte colony-stimulating factor (G-CSF)-mobilized peripheral blood are the three sources of hematopoietic stem cells (HSCs) for transplantation [3]. The level of HSC mobilization is determined by the number of HSCs harvested from the peripheral blood, which is a crucial factor for transplantation. A sufficient number of donor-isolated HSCs secures successful transplantation [4,5]. G-CSF-induced HSC mobilization has become the major technique used for HSCT because it is associated with short hospitalization time, relatively less pain experienced by donors, and quick post-transplant engraftment of white blood cells and platelets [6,7]; however, the efficiency of HSC mobilization is inconsistent, and the underlying mechanisms of mobilization are yet to be investigated [6,8,9]. In addition, current biomarkers are not suitable to indicate HSC mobilization efficiency after the administration of five G-CSF doses. These problems enhance the uncertainty of HSCT.

The high levels of flt3-ligand in plasma prior to G-CSF administration can be used to predict mobilization efficiency [10]. High-cholesterol diets can enhance HSC mobilization from bone marrow to the peripheral blood in mice [11], and cholesterol and low-density lipoprotein cholesterol may serve as key biomarkers for G-CSF-triggered HSC mobilization in humans [12,13]. However, one study demonstrated that the influence of cholesterol level on HSC mobilization is negligible in humans [14]. Furthermore, several single nucleotide polymorphisms of the genes involved in hematopoiesis and cell migration are associated with G-CSF-triggered HSC mobilization [15,16]. Although some factors such as flt3-ligand, cholesterol, low-density lipoprotein cholesterol, and single nucleotide polymorphisms have been reported as possible biomarkers, the specific factors that can effectively and accurately predict HSC mobilization efficiency are yet to be identified.

A high body mass index (BMI) is positively correlated with the extent of HSC mobilization [17,18,19,20,21,22,23]; however, the underlying mechanism is unknown. A high-fat diet and obesity are associated with chronic low-grade systemic inflammation and can induce the secretion of proinflammatory cytokines, such as tumor necrosis factor alpha (TNF-α, interleukin-1 (IL-1), and interferon gamma (IFN-γ) [24,25,26,27,28]. Thus, we hypothesized that a high BMI indicates successful HSC mobilization because of obesity-related chronic inflammation. This study investigated the cosrrelation of BMI and proinflammatory cytokines with G-CSF-triggered HSC mobilization.

## 2. Results

### 2.1. Correlation of BMI with HSC Mobilization

**Figure 1:**
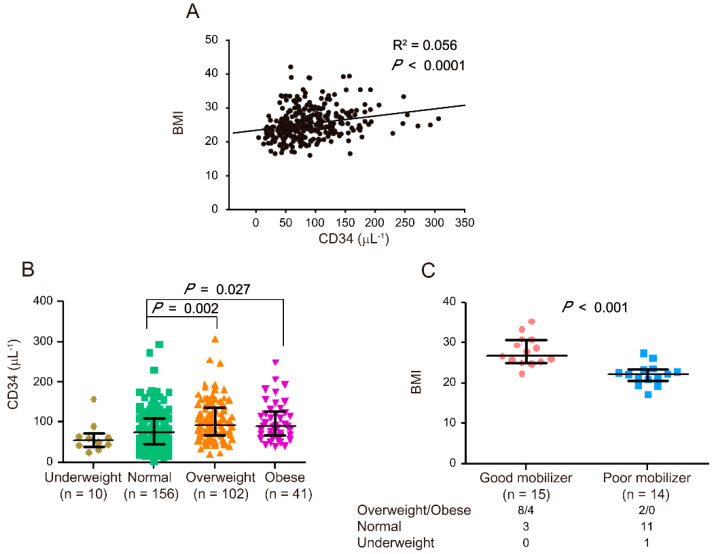
Correlation between body mass index (BMI) and hematopoietic stem cell (HSC) mobilization. Correlation between BMI and CD34^+^ cell counts (μL^−1^) after 5 consecutive days of G-CSF administrations in 309 stem cell donors (R^2^ = 0.056, *p* < 0.0001) (**A**). Correlation between CD34^+^ cell counts (μL^−1^) and BMI in four groups of stem cell donors (**B**). CD34^+^ cell counts in underweight (*n* = 10), normal (*n* = 156), overweight (*n* = 102), and obese (*n* = 41) donors are indicated by a closed circle, square, triangle, and inverted triangle, respectively. Correlation between BMI and HSC mobilization (**C**). BMI is indicated by a closed circle for good mobilizers (*n* = 15) and closed square for poor mobilizers (*n* = 14). The numbers of good and poor mobilizers in the overweight/obese, normal, and underweight groups are indicated. The data are presented as median (Q1, Q3). Graph and statistical significance values were obtained using GraphPad Prism software 5 (Graphstats Technologies, Bengaluru, India).

This retrospective study involved 309 stem cell donors who were administered with G-CSF between August 2015 and January 2018. Data on the age, gender, BMI, and CD34^+^ cell count of all participants were recorded (Table 1). According to previous studies [17,18,19,20,21,22,23], BMI is strongly correlated with CD34^+^ cell count/μL (*p* < 0.0001, Figure 1A) in the peripheral blood. In accordance with the BMI definitions from the World Health Organization, the 309 stem cell donors were categorized into four groups: underweight (BMI < 18.5 kg/m^2^, *n* = 10), normal (18.5 kg/m^2^ ≦ BMI < 25 kg/m^2^, *n* = 156), overweight (25 kg/m^2^ ≦ BMI < 30 kg/m^2^, *n* = 102), and obese (BMI ≧ 30 kg/m^2^, *n* = 41). Compared with the normal donors, the overweight and obese donors exhibited a considerably higher CD34^+^ cell count/μL in their peripheral blood (overweight: *p* *=* 0.002 and obese: *p* = 0.027, Figure 1B). Our results (Supplementary Tables S1 and S2) and previous reports suggested that gender is also an influencing factor that determines the HSC mobilization outcome [17,20,21,23]. Multiple linear regression was further adjusted by age and gender to evaluate the association between BMI and HSC mobilization. Compared with the normal donors, the overweight and obese donors still exhibited higher HSC mobilization (Table 2).

The stem cell donors were categorized as good mobilizers (CD34 ≧ 180/μL, *n* = 15, 4.85%) and poor mobilizers (CD34 ≦ 25/μL, *n* = 14, 4.53%) according to the number of CD34^+^ cells in their peripheral blood after G-CSF administration [4]. The number of good mobilizers in the overweight/obese, normal, and underweight groups was 8/4, 3, and 0, respectively, and that of poor mobilizers was 2/0, 11, and 1, respectively. The good mobilizers exhibited considerably higher BMI than poor mobilizers (*p* < 0.001, Figure 1C). After the adjustment for age and gender, the obtained results were similar to the data without adjustments (Table 3, BMI). Therefore, BMI was significantly and positively correlated with the extent of HSC mobilization.

### 2.2. Correlation of Proinflammatory Cytokine Levels (IFN-γ, IL-22, and TNF-α) with HSC Mobilization

**Figure 2:**
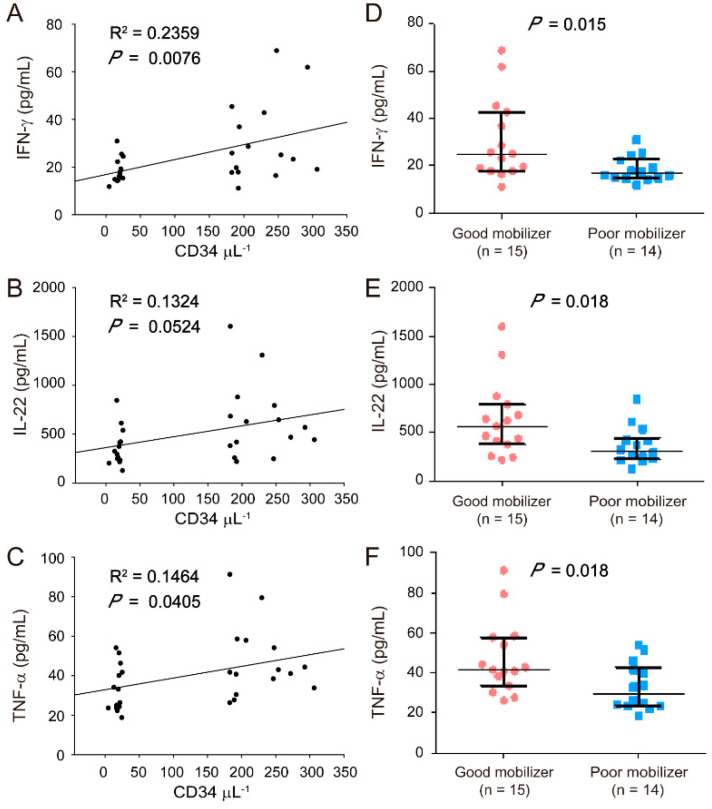
Correlation between proinflammatory cytokines [interferon gamma (IFN-γ), interleukin-22 (IL-22), and tumor necrosis factor-alpha (TNF-α)] and HSC mobilization. Plasma was collected from stem cell donors after 5 consecutive days of G-CSF administrations for ProcartaPlex immunoassay. Correlation between IFN-γ (**A**), IL-22 (**B**), and TNF-α (**C**) levels and CD34^+^ cell counts (μL^−1^) are shown. IFN-γ (**D**), IL-22 (**E**), and TNF-α (**F**) levels are indicated by a closed circle for good mobilizers (*n* = 15) and a closed square (*n* = 14) for poor mobilizers. The data are presented as median (Q1, Q3). Graph and statistical significance values were obtained using GraphPad Prism software.

High BMI may lead to good HSC mobilization because of the release of obesity-related proinflammatory cytokines. Accordingly, the levels of 10 cytokines (IFN-γ, IL-1β, IL-9, IL-10, IL-17A, IL-18, IL-22, IL-23, IL-27, and TNF-α) in the plasma of good mobilizers and poor mobilizers were determined using ProcartaPlex Immunoassay to investigate whether HSC mobilization is associated with the plasma levels of proinflammatory cytokines. The data revealed that the IFN-γ, IL-22, and TNF-α levels were correlated with HSC mobilization (Figure 2A–C) and were considerably higher in the plasma of good mobilizers than in that of poor mobilizers (*p* < 0.05, Figure 2D–F). After the adjustment for age and gender, the obtained results were similar to the data without adjustments (Table 3, IFN-γ, IL-22, and TNF-α). In addition, the differences in the levels of the other cytokines (IL-1β, IL-9, IL-10, IL-17A, IL-18, IL-23, and IL-27) were negligible. However, no association was found between the cytokine levels (IFN-γ, IL-22, and TNF-α) and BMI (Supplementary Figure S1). Therefore, despite the correlation between IFN-γ, IL-22, and TNF-α levels and HSC mobilization in the analyzed population, the good HSC mobilization phenotype associated with high BMI may be not correlated with the secretion of these three proinflammatory cytokines (IFN-γ, IL-22, and TNF-α).

## 3. Discussion

The results clearly showed that BMI and proinflammatory cytokine levels are positively correlated with G-CSF-triggered HSC mobilization. Although the definition of good and poor mobilizers varied among different studies, previous investigators classified poor mobilizers among donors or patients as those in need of apheresis more than once or additional plerixafor treatment to obtain a sufficient amount of HSC for transplantation (>2 × 10^6^ CD34^+^ cells/kg after purification) [29,30]. A linear relationship was also found between peripheral blood CD34^+^ cell count and collected CD34^+^ cell amount after purification [4,5]. In general, the cutoff point of peripheral blood CD34^+^ cell count ranging from less than 10/μL to 50/μL has been set as a threshold to define poor mobilizers [5,14,15,31,32,33,34,35,36]. In the present study, we defined poor mobilizers as having CD34 ≤ 25/μL, a threshold that is within the reported values. To provide a sharp contrast, we defined good mobilizers as having CD34 ≥ 180/μL. All the donors in our study underwent health examination. This phenomenon explains why their cytokine levels were within physiological ranges despite their different BMIs. The higher cytokine levels in good mobilizers than in poor mobilizers further strengthened the notion that BMI, cytokine levels, and HSC mobilization are interrelated. However, our findings should be cautiously interpreted because our analyses were based upon a small cohort of subjects.

Several factors such as gender, cholinesterase, platelet count, red cell count, and mean corpuscular volume are associated with HSC mobilization [19,23,37,38]. Although the correlation between BMI and HSC mobilization has been reported [17,18,19,20,21,22,23,39,40], some contradictions arise. For example, some researchers claimed that BMI has no influence on HSC mobilization [39,40], and others reported the opposite results [17,18,19,20,21,22,23]. This phenomenon indicates that the association between BMI and HSC mobilization remains to be elucidated. Given that the G-CSF doses in the present study were based on the body weight of the stem cell donors, the high HSC mobilization in certain patients might have been caused by the high doses of G-CSF. The largest study (involving 20,884 stem cell donors) to date reported that HSC mobilization was positively correlated with the average daily G-CSF dose in normal and some overweight donors; however, no correlation was observed between daily G-CSF dose and HSC mobilization in obese and severely obese donors who received average daily G-CSF dose higher than 780 and 900 μg, respectively [18]. This finding indicates that BMI is positively correlated with HSC mobilization because of high daily G-CSF doses and other obesity-related factors.

High-fat diets induce chronic inflammation through the secretion of proinflammatory cytokines such as TNF-α, IL-1, IL-2, and IFN-γ [27]. Obesity is associated with chronic low-grade systemic inflammation [24,25,26,41] and the secretion of proinflammatory cytokines (TNF-α, IL-6, and IL-8) by adipocytes or adipose tissue-infiltrating immune cells [28,42,43,44]. In addition to G-CSF, proinflammatory cytokines such as IFN-γ, TNF-α, IL-1, and IL-6 can regulate hematopoiesis during infections [45]. In this study, we observed that the levels of IFN-γ, TNF-α, and IL-22 were considerably higher in the plasma of good mobilizers than in that of poor mobilizers. IFN-γ, which is a type-II IFN, can be produced only by natural killer cells and T cells [46]. It enhances the generation of the earliest CD34^+^ hematopoietic precursor cells in humans and lineage^−^Sca-1^+^c-Kit^+^ (LSK) hematopoietic progenitors in mice [47,48] and the mobilization of HSCs to the spleen during chronic infections [49]. Similar to IFN-γ, TNF-α can regulate HSC proliferation and facilitate hematopoietic engraftment after transplantation [50,51]. TNF-α and IFN-γ have myelosuppressive effect [52,53], which potentially leads to a reduced mobilization effect. However, they also have other effects on stem cells, including improving the immunoregulatory capacity [54] and up-regulating the expression of chemokines and migration [55]. In the present study, IFN-γ and TNF-α possibly exert several actions on the HSCs. Different from other cytokines, IL-22 mainly targets nonhematopoietic epithelial cells and fibroblasts in various tissues, including the lungs, liver, kidney, thymus, pancreas, breast, gut, skin, and synovium, and promotes proliferation and tissue regeneration [56,57]. In this study, we selected 10 proinflammatory cytokines to investigate the relationship of their levels with HSC mobilization. It is also possible that the high levels of pro-inflammatory cytokines that we observed are not the cause of the high mobilization of CD34^+^ cells. These two events (CD34^+^ cells mobilization and release of inflammatory cytokines) could not be linked by a cause-effect relationship. Indeed, these two events can both be caused by a third factor, hitherto not studied, which is able to cause both the release of inflammatory cytokines and CD34^+^ cells mobilization. Future research should focus on the correlation of other cytokines with HSC mobilization; the nature of influence (direct or indirect) of IFN-γ, TNF-α, and IL-22 on HSC proliferation or mobilization; and the association of high BMI-induced good HSC mobilization with the release of obesity-related proinflammatory cytokines.

In a recent study, short-term fat-free diet enhanced HSC mobilization efficiency in mice [58]. Fat-free diets can help reduce the levels of ω3-polyunsaturated fatty acids, such as eicosapentaenoic acid in the bone marrow. Eicosapentaenoic acid binds to bone marrow myeloid cells through β1/β2-adrenergic receptors and up-regulates peroxisome proliferator-activated receptor δ. It then activates angiopoietin-like protein 4, which in turn inhibits vascular permeability and HSC mobilization [58]. ω3-Polyunsaturated fatty acids exert anti-inflammatory effects and can reduce the levels of inflammatory cytokines, such as IL-1, IL-6, and TNF-α, and mitigate adipose tissue inflammation in animal models of obesity [59,60,61,62]. Whether the results obtained from mice can be generalized to humans remains unclear. Nevertheless, current and previous findings [58,59,60,61,62] indicate that proinflammatory cytokines may enhance HSC mobilization. Of note, previous studies have reported that G-CSF that was used to promote the mobilization of stem cells to the peripheral blood could cause adverse effects such as idiopathic pneumonia [63] and capillary leak syndrome [64]. These adverse consequences may be related to the high levels of circulating inflammatory cytokines during the mobilization.

This study demonstrated that BMI is correlated with HSC mobilization [17,18,19,20,21,22,23]. We also found that the levels of three proinflammatory cytokines (IFN-γ, IL-22, and TNF-α) are considerably increased in the plasma of good mobilizers compared with those in the plasma of poor mobilizers. Further investigation is required to determine whether high BMI-induced good HSC mobilization is associated with the release of obesity-related proinflammatory cytokines. The mechanism through which proinflammatory cytokines enhance HSC mobilization must also be further explored.

## 4. Materials and Methods

### 4.1. Stem Cell Donors

A total of 309 healthy stem cell donors voluntarily participated in this study between August 2015 and January 2018 and provided their written informed consent. The donors’ personal data on gender, body weight, body height, BMI (kg/m^2^), and number of CD34^+^ cells and residual samples were obtained in compliance with the protocols approved by the Institutional Review Board of Buddhist Tzu Chi General Hospital (approval ID: IRB099-131 and IRB104-152-A).

### 4.2. Human Sample and Plasma Collection

G-CSF (Filgrastim, Kirin Brewery Co., Tokyo, Japan) was injected subcutaneously at a dosage of 10 μg/kg per day for 5 consecutive days [23]. The number of CD34^+^ cells was analyzed at the Department of Laboratory Medicine (Hualien Tzu Chi Medical Center) after the last G-CSF injection [23]. After G-CSF administration, 50 μL of peripheral blood was incubated with phycoerythrin-conjugated CD34 antibody and analyzed using flow cytometry (Becton-Dickinson, San Jose, CA, USA). The residual specimens (1–2 mL) were diluted by twofold dilution with phosphate-buffered saline and layered on 5 mL of Ficoll-Paque PLUS (GE Healthcare, Chicago, IL, USA). The plasma fractions were collected after centrifugation (400× *g* for 40 min) at room temperature.

### 4.3. Measurement of Cytokines

The plasma specimens were diluted by twofold dilution with phosphate-buffered saline, and the levels of several cytokines (IFN-γ, IL-1 β, IL-9, IL-10, IL-17A, IL-18, IL-22, IL-23, IL-27, and TNF-α) were measured using a custom-made ProcartaPlex Immunoassay (Thermo Fisher Scientific, Waltham, MA, USA).

### 4.4. Statistical Analysis

The data are presented as mean ± standard deviation or median (Q1, Q3) depending on their distribution (normal or not). Statistical analyses were performed using Microsoft Office Excel 2003 (Redmond, WA, USA) and GraphPad Prism 5 software (Graphstats Technologies, Bengaluru, India). Graphs were plotted using SigmaPlot 10.0 (Systat Software, San Jose, CA, USA) and GraphPad Prism 5 software. Shapiro–Wilk test (*n* < 50) was adopted to examine whether the data were normally distributed. Continuous variables with non-normal distribution were compared using either Wilcoxon rank-sum test in the cases of two independent groups or Kruskal–Wallis test followed by a post-hoc test in the case of four independent groups and were presented as median with interquartile range (IQR). After the adjustment for age and sex, multiple linear regression was used to evaluate the association between BMI and HSC mobilization and between HSC mobilization and proinflammatory cytokines. *p* values less than 0.05 were considered statistically significant.

## Figures and Tables

**Table 1:** Characteristics of study subjects.

Characteristic	Range	Number
* Age	34.3 ± 7.7	
Gender		
Female		106
Male		203
* Body mass index (BMI) (kg/m^2^)	24.7 (22.5, 27.8)	
BMI < 18.5		10
18.5 ≤ BMI < 25		156
25 ≤ BMI < 30		102
BMI ≥ 30		41
* CD34^+^ cell count (µL^−1^)	80.4 (53.3, 120.3)	
CD34^+^ ≤ 25		14
25 < CD34^+^ < 180		280
CD34^+^ ≥ 180		15

* Data are presented as mean ± standard deviation or median (Q1, Q3).

**Table 2:** Correlation of BMI with HSC mobilization after the adjustment for age and gender (*n* = 309).

	Crude		Adjusted	
	β (95% CI)	*p*-Value	β (95% CI)	*p*-Value
Age	0.16 (−0.57, 0.89)	0.670	−0.10 (−0.80, 0.61)	0.790
Gender	28.39 (17.00, 39.78)	<0.001 *	26.25 (14.85, 37.65)	<0.001 *
(Male vs. Female)				
BMI group	-	-	-	-
Normal	Ref.	Ref.	Ref.	Ref.
Underweight	−17.94 (−49.32, 13.44)	0.261	−15.52 (−45.89, 14.85)	0.315
Overweight	21.63 (9.38, 33.88)	0.001 *	16.99 (4.88, 29.11)	0.006 *
Obese	22.55 (5.67, 39.43)	0.009 *	18.13 (1.62, 34.65)	0.032 *

Data are presented as β (95% CI). * *p*-value < 0.05 was considered statistically significant after test. Abbreviations: BMI = body mass index; Ref. = reference.

**Table 3:** Correlation of BMI, IFN-γ, IL-22, and TNF-α with HSC mobilization after the adjustment for age and gender (*n* = 29).

	BMI		IFN-γ		IL-22		TNF-α	
	β (95% CI)	*p*-Value	β (95% CI)	*p*-Value	β (95% CI)	*p*-Value	β (95% CI)	*p*-Value
Age	0.18 (0.02, 0.34)	0.026 *	−0.20 (−0.96, 0.56)	0.589	−0.38 (−18.11, 17.36)	0.965	−0.24 (−1.11, 0.63)	0.573
Gender (Male vs. Female)	0.15 (−2.62, 2.92)	0.914	−4.47 (−17.86, 8.92)	0.497	−252.47 (−564.45, 59.51)	0.108	−16.04 (−31.35, −0.74)	0.041 *
Group (Good vs. Poor)	5.42 (2.71, 8.14)	<0.001 *	15.9 (2.76, 29.03)	0.020 *	462.07 (155.92, 768.22)	0.005 *	24.97 (9.95, 39.99)	0.002 *

Data are presented as β (95% CI). * *p*-value < 0.05 was considered statistically significant after test. Abbreviations: BMI = body mass index; IFN = interferon; IL = interleukin; TNF = tumor necrosis factor; HSC = hematopoietic stem cell.

## Data Availability

All data relevant to the study are included in the article. The original data are available from the corresponding author upon reasonable request.

## Supplementary Materials

The following supporting information can be downloaded at: https://www.mdpi.com/article/10.3390/jcm11144169/s1, Figure S1: Correlation of the levels of cytokines (IFN-γ, IL-22, and TNF-α) and body mass index (BMI); Table S1: Comparison of characteristics among different BMI groups; Table S2: Comparison of characteristics between good and poor mobilizers.

## Abbreviations

HSCT	hematopoietic stem cell transplantation
G-CSF	granulocyte colony-stimulating factor
HSCs	hematopoietic stem cells
BMI	body mass index
TNF-α	tumor necrosis factor alpha
IL	interleukin
IFN-γ	interferon gamma
