# Supplementary material for: Correlation of Body Mass Index and Proinflammatory Cytokine Levels with Hematopoietic Stem Cell Mobilization
Source: J Clin Med. 2022 Jul 18;11(14):4169. doi: 10.3390/jcm11144169 (PMC9317243; doi:10.3390/jcm11144169)
Supplement: Supplementary file 1 [file jcm-11-04169-s001.zip › jcm-1723588-supplementary.pdf]

# Supplementary Materials

# Correlation of body mass index and proinflammatory cytokine levels with hematopoietic stem cell mobilization

**Tso-Fu Wang [1,2,3,†], Yu-Shan Liou [4,†], Hsin-Hou Chang [4], Shang-Hsien Yang [2,3,5], Chi-Cheng Li [1,6], Jen-Hung Wang [7] and Der-Shan Sun [4,*]**

1 Department of Hematology and Oncology, Hualien Tzu Chi Hospital, Buddhist Tzu Chi Medical Foundation, Hualien 97002, Taiwan; tfwang@tzuchi.com.tw (T.-F.W.); kevinlcc1234@gmail.com (C.-C.L.)
2 Department of Medicine, College of Medicine, Tzu Chi University, Hualien 97004, Taiwan; hermann_yang@tzuchi.com.tw
3 Buddhist Tzu Chi Stem Cells Center, Hualien Tzu Chi Hospital, Buddhist Tzu Chi Medical Foundation, Hualien 97002, Taiwan
4 Department of Molecular Biology and Human Genetics, College of Medicine, Tzu Chi University, Hualien 97004, Taiwan; az0922663053@gmail.com (Y.-S.L.); hhchang@mail.tcu.edu.tw (H.-H.C.)
5 Department of Pediatrics, Hualien Tzu Chi Hospital, Buddhist Tzu Chi Medical Foundation, Hualien 97002, Taiwan
6 Center of Stem Cell & Precision Medicine, Hualien Tzu Chi Hospital, Buddhist Tzu Chi Medical Foundation, Hualien 97002, Taiwan
7 Department of Medical Research, Hualien Tzu Chi Hospital, Buddhist Tzu Chi Medical Foundation, Hualien 97002, Taiwan; jenhungwang2011@gmail.com
* Correspondence: dssun@mail.tcu.edu.tw; Tel.: +886-3-8565301 (ext. 2681); Fax: +886-3-8561422
† These authors contributed equally to this work.

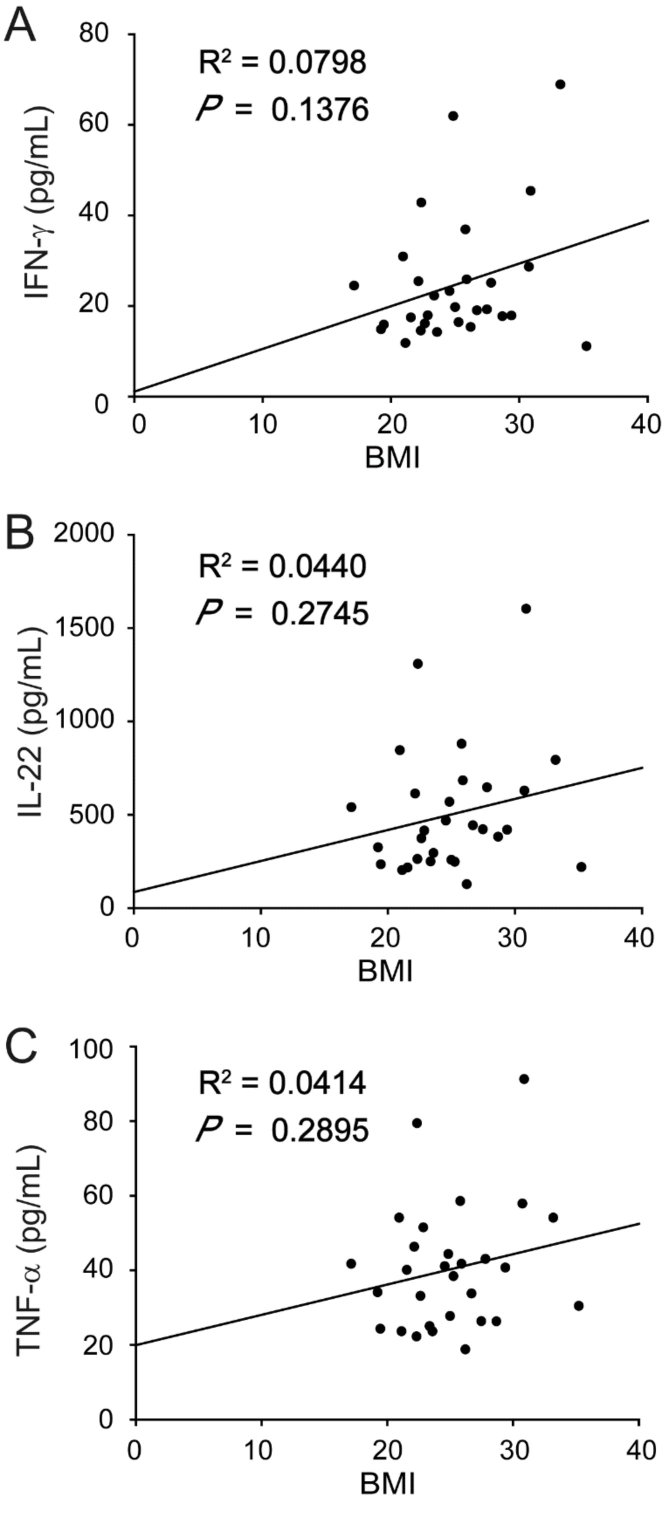


**Figure S1.** Correlation of the levels of cytokines (IFN-$\gamma$, IL-22, and TNF-$\alpha$) and body mass index (BMI).

**Table S1.** Comparison of characteristics among different BMI group.

| | Underweight | Normal | Overweight | Obese | Total | *p*-Value |
|---|---|---|---|---|---|---|
| No. | 10 | 156 | 102 | 41 | 309 | |
| Age | 34.2 ± 5.5 | 33.5 ± 8.3 | 35.8 ± 7.2 | 33.9 ± 6.0 | 34.3 ± 7.7 | 0.140 |
| Gender | - | - | - | - | - | 0.015 * |
| Female | 5 (50.0%) | 92 (59.0%) | 79 (77.5%) | 27 (65.9%) | 203 (65.7%) | |
| Male | 5 (50.0%) | 64 (41.0%) | 23 (22.5%) | 14 (34.1%) | 106 (34.3%) | |
| CD34 | 56.1 (40.9, 71.5) | 74.2 (46.2, 108.9) | 92.2 (67.0, 136.5) | 90.8 (68.7, 128.2) | 80.4 (53.3, 120.3) | <0.001 * |

Data are presented as number or mean ± standard deviation or median (Q1, Q3). * $p$-value < 0.05 was considered statistically significant after test. Abbreviations: BMI = body mass index.

**Table S2.** Comparison of characteristics between good and poor mobilizers.

| | Good Mobilizer | Poor Mobilizer | Total | *p*-Value |
|---|---|---|---|---|
| No. | 15 | 14 | 29 | |
| Age | 33.6 ± 7.6 | 36.1 ± 6.3 | 34.9 ± 6.9 | 0.365 |
| Gender | - | - | - | 0.002 * |
| Female | 12 (80.0%) | 3 (21.4%) | 15 (51.7%) | |
| Male | 3 (20.0%) | 11 (78.6%) | 14 (48.3%) | |
| BMI | 26.7 (25.0, 30.7) | 22.3 (20.6, 23.5) | 24.9 (22.3, 27.7) | <0.001 * |

Data are presented as number or mean ± standard deviation or median (Q1, Q3). *$p$-value < 0.05 was considered statistically significant after test.
